# Upregulation of TSHR, TTF-1, and PAX8 in Nodular Goiter Is Associated with Iodine Deficiency in the Follicular Lumen

**DOI:** 10.1155/2016/2492450

**Published:** 2016-07-25

**Authors:** Huibin Huang, Lijun Chen, Bo Liang, Huiyao Cai, Qingyan Cai, Yaxiong Shi

**Affiliations:** ^1^Department of Endocrinology, The Second Affiliated Hospital of Fujian Medical University, Quanzhou, Fujian 362000, China; ^2^Postgraduate School, The Second Clinical Medical College of Fujian Medical University, Quanzhou, Fujian 362000, China

## Abstract

*Objective*. It has been testified that iodine regulates thyroid function by controlling thyroid-restricted genes expression and is closely related to diffuse goiter and thyroid dysfunction. However, the effects of follicular lumen iodine, the main form of iodine reserve in the body, on thyroid-restricted genes in nodular goiter are poorly understood. In this study, correlations between follicular lumen iodine and the expressions of thyroid stimulating hormone receptor (TSHR), its transcription factors TTF-1, and PAX8 in nodular goiter were investigated.* Patients*. In this study, 30 resection specimens clinically histopathologically confirmed to have nodular goiter and 30 normal thyroid specimens from adjacent tissues of nodular goiter are used.* Measurement*. Western blot immunohistochemistry was performed to assay TSHR, TTF-1, and PAX8 in thyrocytes of nodular goiter as well as in extranodular normal thyroid tissues. Meanwhile, follicular lumen iodine of both nodular goiter and extranodular normal thyroid tissues was detected as well.* Results*. The TSHR, TTF-1, and PAX8 in nodular goiter were significantly higher than those in the controls. The iodine content in nodular goiter was significantly lower than those in control tissues.* Conclusion*. Upregulation of TSHR, TTF-1, and PAX8 is associated with low follicular lumen iodine content in nodular goiter.

## 1. Introduction

Iodine is a key ingredient in the synthesis of thyroid hormones and also a major factor in the regulation of thyroid function [1, 2]. At the organ level, small changes in the absorption of iodine can allow the thyroid to adjust its response to TSH stimulation and stabilize thyroid function. Long-term severe iodine deficiency will cause diffuse goiter and thyroid dysfunction [3]. On the other hand, large dose of iodine intake blocks iodine organification and inhibits the synthesis and release of thyroid hormones. This is called the “Wolff-Chaikoff” effect [4, 5]. At the cellular level, iodine has been shown to inhibit multiple signaling pathways of thyroid cells, including CAMP and PIP2. Iodine not only reduces the expression of thyroid-specific proteins such as NIS but also has been shown to alter the TSH/TSHr signaling pathway [6–8]. At the follicular level, iodine in follicular lumen, namely, iodinated thyroglobulin (iodinated TG), is the main form of iodine reserve in the body. It has been demonstrated that iodinated TG in the follicular lumen can suppress thyroid function and that the suppression takes place through downregulation of TSHR expression in thyroid follicular cells [9–11].

TSHR belongs to a class of G-protein-coupled receptors and it is expressed on the surface of thyroid cells. It is regulated by iodine and also by the thyroid-specific transcription factors TTF-1 and PAX8 [12, 13]. TSH binds with TSHR to deliver extracellular signal and regulates thyroid function and growth of thyroid cells [14, 15]. Overexpression of local follicular TSHR may overstimulate this part of thyroid tissue to form hyperplasia, ultimately leading to the formation of nodules. Nodular goiter is a proliferative disease. The expression patterns of TSHR, TTF-1, and PAX8 in the diseased tissues and the regulation of their protein expression by follicular lumen iodine have not yet been elucidated. To preliminarily study the pathogenesis of nodular goiter, the association of the follicular lumen iodine with TSHR, TTF-1, and PAX8 expression in nodular goiter lesions was here investigated.

## 2. Materials and Methods

### 2.1. Patients

After obtaining the approval of the institutional research ethics board, 30 resection specimens clinically histopathologically confirmed to have nodular goiter and 30 normal thyroid specimens from adjacent tissues of nodular goiter are used as the negative control were collected. All the patients with course of the disease 0.5–10 years and age of 40.5 ± 14 years have euthyroid function and were not treated with medicine.

### 2.2. Western Blot Analysis

All protein samples were prepared as follows: 0.5 g of tissue was frozen in liquid nitrogen, ground to yield tissue powder, and then suspended in ice-cold RIPA lysis buffer (ABcom Co., Ltd., Shanghai, China). The suspension was then sonicated for 1 min at 0–8°C and centrifuged at 8000 ×g for 30 min. The protein concentration was determined by BCA (Sigma, Shanghai, China) assay. The lysates were then separated electrophoretically in 12% polyacrylamide gels and transferred onto PVDF membranes (Beijing Biosynthesis Biotechnology Co., Ltd., Beijing, China). The membranes were blocked for 1.5 h at room temperature in 5% nonfat milk and incubated overnight at 4–8°C with TSHR, TTF-1, and PAX8 antibodies (the same as those used in immunohistochemistry), respectively. After the triple wash in Tris buffered saline with tween (TBST) for 30 min, the membranes were incubated with HRP-conjugated secondary antibodies for 45 min and then triple washed again in TBST. Immune reactive bands were revealed using ECL detection system. In the negative group, 2% BSA was used instead of the primary antibodies. *β*-actin (Beijing Biosynthesis Biotechnology Co., Ltd., Beijing, China) was also detected as an internal control. The X-ray film was scanned, and the band density was calculated using ImageJ 1.45 software.

### 2.3. Immunohistochemistry Analysis

The tissues were fixed in 10% formalin and embedded in paraffin. Sections (4 mm) were taken from the tissues, affixed to 3-aminopropyl triethoxysilane-coated slides, and air-dried overnight at 37°C. After dewaxing and antigen retrieval, endogenous peroxidase was quenched with 3% hydrogen peroxide for 5 min. After blocking with goat serum, the slides were incubated for 30 min at 37°C with mouse anti-TSHr (at 1 : 100 dilution), rabbit anti-TTF-1 (at 1 : 300 dilution), and rabbit anti-PAX8 (at 1 : 150 dilution) polyclonal antibody (Beijing Biosynthesis Biotechnology Co., Ltd., Beijing, China), respectively. All procedures were performed with a diaminobenzidine (DAB) detection kit (Beijing Biosynthesis Biotechnology Co., Ltd., Beijing, China) following the standard protocol. After washing, all slides were counterstained with haematoxylin-eosin for histological evaluation. For the negative control, the step on incubation with a primary antibody was omitted. The sections were examined under a microscope.

### 2.4. Immunohistochemical Evaluation

The sections were observed randomly in 10 high-power fields (not follicular lumen area). The results were scored semiquantitatively. The intensity of staining was scored as no staining (0), weak staining (1), moderate staining (2), or strong staining (3). The percentage of stained thyroid cells was scored as no stained cells (0), staining in 25% of the cells (1), staining in 25–50% of the cells (2), staining in 50–75% of the cells (3), or staining in more than 75% of the cells (4). The final result of each section was evaluated by averaging the sum of the intensities and percentages of the stained thyroid cells. The average was then scored as 0–2 (negative), 3-4 (positive), and 5–7 (strongly positive).

### 2.5. Follicular Lumen Iodine Detection

Each protein suspension was taken and the iodine concentration of suspension was then determined by arsenic-cerium catalytic spectrophotometry (Venter Health Science and Technology Co., Ltd., Hubei, China) according to standard procedures of manufacturer' instructions. The follicular lumen iodine content was defined as iodine (*μ*g)/tissues (mg).

### 2.6. Statistical Analysis

SPSS19 software was employed for statistical analysis. Student's *t*-test was used to determine the differences of the western blot whereas the Fisher exact test was used to determine the differences in positive-staining rate. In addition, the Wilcoxon rank sum test was used for determining the differences in stain results and the Spearman rank coefficient was used for determining the correlation between the parameters. Statistical differences were identified and *P* value 0.05 was considered significant.

## 3. Results

### 3.1. Western Blot Analysis of TSHR, TTF-1, and PAX8 Protein and Evaluation of Follicular Lumen Iodine Content in Nodular Goiter

**Figure 1:**
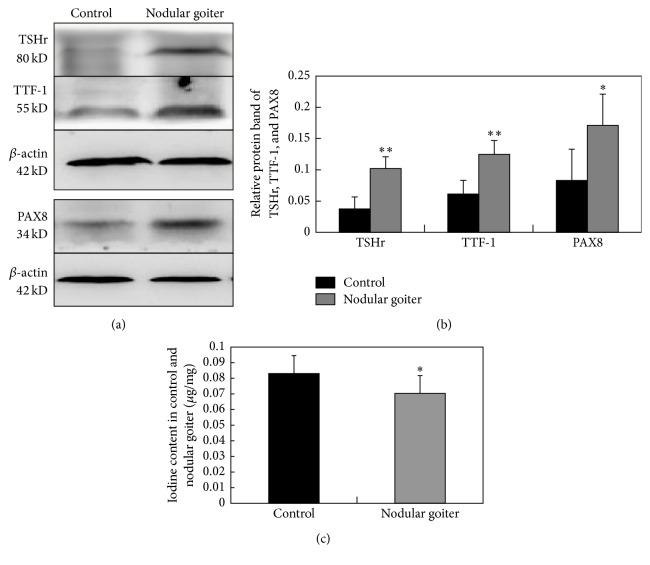
Western blot assay for the expressions of TSHR, TTF-1, and PAX8 protein in nodular goiter and the control; *β*-actin is also detected as an internal control. The expressions of TSHR, TTF-1, and PAX8 in nodular goiter are significantly higher than those in the control. *n* = 10 (a, b). The iodine content (*μ*g/mg) in nodular goiter was significantly lower than those in control tissues. *n* = 30 (c). ^*∗*^ *P* < 0.05; ^*∗∗*^ *P* < 0.01.

Western blot was performed to determine the expression of TSHR, TTF-1, and PAX8 in 10 nodular goiter and 10 control thyroid samples. in addition to the increase in TSHR expression, the expressions of TTF-1 and PAX8 in nodular goiter lesions were also significantly higher than those in control tissues (Figures 1(a) and 1(b)). Follicular lumen iodine concentration was also determined by arsenic-cerium catalytic spectrophotometry in 30 nodular goiter and 30 control thyroid samples. The iodine content (*μ*g/mg) in nodular goiter was significantly lower than those in control tissues (Figure 1(c)).

### 3.2. Immunohistochemistry Analysis of TSHR, TTF-1, and PAX8 Expression in Nodular Goiter

**Figure 2:**
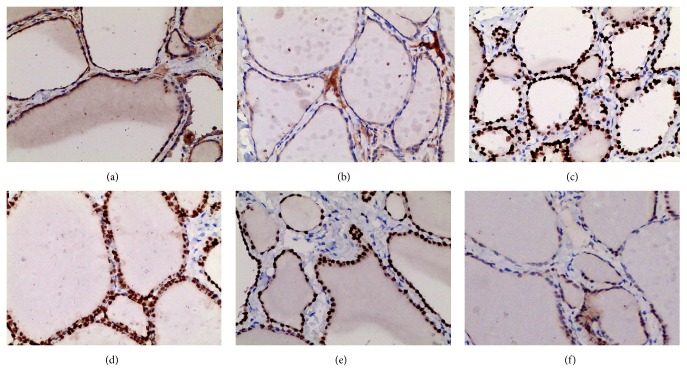
Immunohistochemical analysis of the expressions and localization of TSHR, TTF-1, and PAX8 in nodular goiter and the control. The high expression of TSHR in nodular goiter is shown in (a), the moderate expression of TSHR in the controls is shown in (b); the upregulation of TTF-1 in nodular goiter is shown (c), and the moderate expression of TTF-1 in the controls is shown in (d). The high expression of PAX8 in nodular goiter is shown in (e), and the moderate expression of PAX8 in the controls is shown in (f).

Immunohistochemistry was also performed to determine TSHR, TTF-1, and PAX8 expression in 30 nodular goiter and 30 control thyroid samples. TSHR was localized in the basolateral membrane, and the immune reactivity of TSHR in nodular goiter was significantly higher than those in the control (Figures 2(a) and 2(b) and Table 1). TTF-1 and PAX8 were confined to the nucleus of the thyrocytes, and both types of immune reactivity in nodular goiter were significantly higher than those in the control (Figures 2(c), 2(d), 2(e), and 2(f) and Tables 2 and 3).

### 3.3. The Correlation between the Parameters of the Follicular Lumen Iodine Content, TSHr, TTF-1, and PAX8

The Spearman rank coefficient was performed to determine the correlation among the parameters of TSHR, TTF-1, PAX8, and iodine in 30 nodular goiter and control samples. The results showed that iodine content in follicular lumen of nodular goiter was less than that in control group; moreover, the content of iodine in follicular lumen was found to be negatively correlated with TSHR (*r*_*s*_ = −0.857; *P* < 0.01), TTF-1 (*r*_*s*_ = −0.805; *P* < 0.01), and PAX8 (*r*_*s*_ = −0.652; *P* < 0.05) expression in nodular goiter.

## 4. Discussion

Nodular goiter is a complex disease, caused by genetic and environmental interaction. Environmental factors include iodine nutrition [16], smoking, estrogen, obesity, age, and gender [17, 18]. Genetic factors are mainly related to the polymorphism or mutation of thyroid-specific genes, including TSHR, TPO, TG, and NIS [19, 20]. Epidemiological studies have suggested that iodine deficiency is the major risk factor of nodular goiter, which may be related to thyroid dysfunction caused by iodine deficiency [18] and subsequent chronic thyroid stimulation caused by enhanced TSH level and its binding to TSHR.

Thyroid follicles are heterogeneous [21]. Individual thyroids may differ considerably in response even when stimulated by the same concentration of TSH. Some follicles respond normally to TSH, but others may form nodular hyperplasia due to excessive stimulation of TSH. It has been found that the heterogeneity of thyroid follicles was correlated with different levels of TSHR expression on the surface of follicles. Follicles rich in expression of TSHR showed a strong response to TSH/TSHR stimulation [22]. On the contrary, the reaction was weak. Western blot analysis showed TSHR protein expression to be significantly greater in nodular goiter lesions than in the adjacent normal thyroid tissues. Likewise, immunohistochemical staining also showed there to be more TSHR staining was stronger in the basement membranes of cells in lesions than in normal tissues, indicating that TSHR is highly expressed in nodular goiter lesions. This shows that TSH stimulation is stronger in nodular goiter lesions than in normal tissues. This leads to excessive proliferation of follicular cells and is one of the causes of nodule formation [23].

TSHR is G protein-coupled receptor, mainly located in the basement membrane of thyroid epithelial cells. It facilitates the regulation of thyroid cells by TSH from the central pituitary [24]. TSHR expression is mainly regulated by the thyroid-specific transcription factors TTF-1 and PAX8. TTF-1 and PAX8 bind to the regulatory region of the TSHR gene, promoting the expression of TSHR and increasing TSH/TSHR stimulation signals [25, 26]. In addition, TSHR is also regulated by iodine in the follicular lumen [9, 27]. Results showed that, in addition to the increase in TSHR expression, nodular goiter lesions also expressed significantly more TTF-1 and PAX8 than normal thyroid tissues. TSHR expression in nodular goiter lesions may be associated with upregulated expression of transcription factors TTF-1 and PAX8. It may also be associated with the feedback regulation of follicular iodine.

The thyroid is the body's main storage site of iodine, and thyroid iodine is mainly stored in the thyroid follicles [28]. In this way, iodinated TG represents iodine in the follicular lumen. Iodinated TG is the macromolecular precursor of thyroid hormones. Iodinated TG in the follicular lumen reflects not only the size of the thyroid hormone reserve but also that of the iodine reserve [29]. In this way, they have important feedback effects on thyroid function. Studies have shown that exogenous TG could inhibit the expression of thyroid-specific genes in monolayer cells in vitro [21, 30, 31]. In addition, results also showed that the iodinated TG in follicular lumen reduced the sensitivity of thyroid follicles to TSH from the pituitary by inhibiting the expression of TSHR, eventually leading to downregulated expression of thyroid-specific genes NIS, TPO, and TG [10, 32]. The discovery also explained the heterogeneity of thyroid follicles and indicated that when all thyroid follicles in the body had the same concentrations of iodine and TSH, there were significant differences in the sizes and functions of individual follicles. Different levels of iodinated TG are associated with different levels of thyroid sensitivity TSH. Negative feedback regulation of iodinated TG in the follicular lumen may be an important cause of thyroid follicular heterogeneity in the body [11]. The present work showed nodular goiter lesions to contain significantly less iodine than normal thyroid tissue, and iodine content was found to be negatively correlated with TSHr expression, suggesting that iodine in follicular lumen inhibits expression of TSHr and low iodine status in the follicular lumen of nodular goiter promotes upregulation of TSHR expression.

It remains unclear whether low iodine in follicular lumen upregulated TSHR expression directly or through induction of TTF-1 and PAX8. The current study showed that low iodine status in nodular goiter was associated with high levels of expression of TTF-1 and PAX8. TTF-1 and PAX8 are positive transcription factors in the expression of thyroid-specific proteins TSHR, TPO, TG, and NIS. Increased expression of these factors was found to promote increased synthesis of TSHR. They not only strengthened the iodine absorption of thyroid epithelial cells, iodine organification, polyiodide, and other reactions, enhancing thyroid hormone synthesis and iodine accumulation, but also stimulated the hyperplasia of thyroid gland, which also plays a role in the formation of thyroid nodules.

Follicular iodinated TG is a macromolecular glycoprotein and cannot freely penetrate the thyroid cells. The mechanism by which regulation of iodinated TG in turn regulates TSHR, TTF-1, and PAX8 is still unclear. One previous report has suggested that TG binds to sialic acid receptors in the apical membrane of follicular lumen [32–34]. Further study is necessary to determine whether sialic acid receptor mediates the regulation of TG.

The incidence of nodular goiter has increased significantly in recent years. This study demonstrated the negative feedback regulation of iodine and iodinated TG in the expression of TSHR, TTF-1, and PAX8 in the follicular lumen, which further regulates the response heterogeneity of thyroid follicles to TSH stimulation. The current findings have important implications for further studies of the pathogenesis of these diseases.

## Figures and Tables

**Table 1:** Immunohistochemistry detecting TSHR in control and nodular goiter groups.

Group	Staining result					
	Cases (*n*)	Negative	Positive	Strong positive	Positive rate (%)	Strong positive rate (%)
Control	30	0	30	8	100	26.7^*∗*^
Nodular goiter	30	0	30	26	100	86.7^*∗*^

^*∗*^ *P* < 0.01.

**Table 2:** Immunohistochemistry detecting TTF-1 in control and nodular goiter groups.

Group	Staining result					
	Cases (*n*)	Negative	Positive	Strong positive	Positive rate (%)	Strong positive rate (%)
Control	30	0	30	12	100	40^*∗*^
Nodular goiter	30	0	30	24	100	80^*∗*^

^*∗*^ *P* < 0.01.

**Table 3:** Immunohistochemistry detecting PAX8 in control and nodular goiter groups.

Group	Staining result					
	Cases (*n*)	Negative	Positive	Strong positive	Positive rate (%)	Strong positive rate (%)
Control	30	0	30	11	100	36.7^*∗*^
Nodular goiter	30	0	30	23	100	76.7^*∗*^

^*∗*^ *P* < 0.01.
